# A Treatise on the Practice of Medicine

**Published:** 1876-12

**Authors:** 


A TREATISE ON THE PRACTICE OF MEDICINE. By John Syer Bristowe, M. D., London, F. R. C. P., Physician to St. Thomas Hospital, etc., etc. Edited with notes by James H. Hutchinson, M.D., one of the attending Physicians to the Pennsylvania Hospital, etc. Philadelphia: Henry C. Lea. 1876.

This work is comprised in one volume of 1050 pages, and is exhaustive in the consideration of certain classes of disease. About sixty pages, for instance, are occupied with skin diseases, and over one hundred in the consideration of disturbance connected with the respiratory organs. On the other hand, while female diseases are alluded to by the American editor, in his preface to the work, as an important and rare feature in works of the kind, we must say that the limited scope given to this subject will not impress the reader as likely to lessen the necessity for consulting works more exhaustive and thorough on diseases peculiar to females. The work, taken altogether, although retaining much exploded doctrine of causation and treatment of disease, is equal, and in some respects superior, to other books of the kind. The notes added by the American editor relieve the work of some objectional features, but the fear of disturbing too much a work which has, in former editions, had "a fine run," seriously retards progress in medicine. As a text book this is suited to the wants of students, and will doubtless find general favor.

